# Intratracheal myiasis followed by tracheal-esophageal fistula: report of a rare case and literature review

**DOI:** 10.1186/s12879-019-4679-7

**Published:** 2019-12-17

**Authors:** Wendi Huang, Chao Zeng, Weidong Song, Ping Xu

**Affiliations:** Departments of Respiratory and Critical Care Medicine, Peking University Shenzhen Hospital, Lianhua Road, Shenzhen, 518036 Guangdong China

## Abstract

**Background:**

To enhance awareness of the clinical features and prevention of endotracheal myiasis.

**Case presentation:**

A case of intratracheal myiasis is reported. A 61-year-old male patient with a history of laryngectomy was admitted to hospital due to tracheostomal hemorrhage of 3 h duration. Intratracheal myiasis was confirmed by bronchoscopy, and the patient underwent bronchoscopic intervention, which was complicated by a tracheal-esophageal fistula and resolved by endotracheal stenting. Twenty months after stent placement, the fistula had not healed.

**Conclusion:**

Intratracheal myiasis has serious complications and is difficult to treat. For post-tracheostomy patients, healthcare providers and caregivers should pay attention to the care and monitoring of wounds and maintenance of a tidy, clean living environment to prevent intratracheal myiasis.

## Background

Myiasis is the invasion of dipterous insect larvae into tissues. The larvae can invade human skin, gangrenous tissue, and natural cavities. Myiasis of the trachea is very rare with only a few cases previously reported. Furthermore, intratracheal myiasis followed by a tracheal-esophageal fistula has not, to the best of our knowledge, been reported previously [1–5].

## Case presentation

A 61-year-old man from Shenzhen presented with tracheostomal hemorrhage of 3 h duration. One year previously, the patient had been diagnosed with laryngeal squamous cell carcinoma by electronic laryngoscopy, and had undergone partial laryngectomy and tracheotomy under general anesthesia. Following these procedures, he was discharged and recovered well. The tracheotomy was maintained after discharge. The patient and his family cleaned the tracheal cannula themselves which may have resulted in inadequate care. The patient visited our hospital due to tracheostomal hemorrhage of 3 h duration.

Physical examination at admission showed peri-tracheostomal redness and correct positioning of the tracheal cannula. Bloody secretions were seen in the dressing. Electronic laryngoscopy showed postoperative changes in the glottic area, with scar formation; the airway was patent. The results of a complete blood count were 9.58 × 10^9^/L white blood cells, 74.4% neutrophils, and 1.0% eosinophils. After admission, the tracheal cannula was removed, and the surface of the cannula was dirty. The stomal tissue was red and swollen. On the day after removal of the tracheal cannula, the patient coughed out maggot larvae through the tracheostomy, and several larvae were found in the peri-stomal area.

**Fig. 1:**
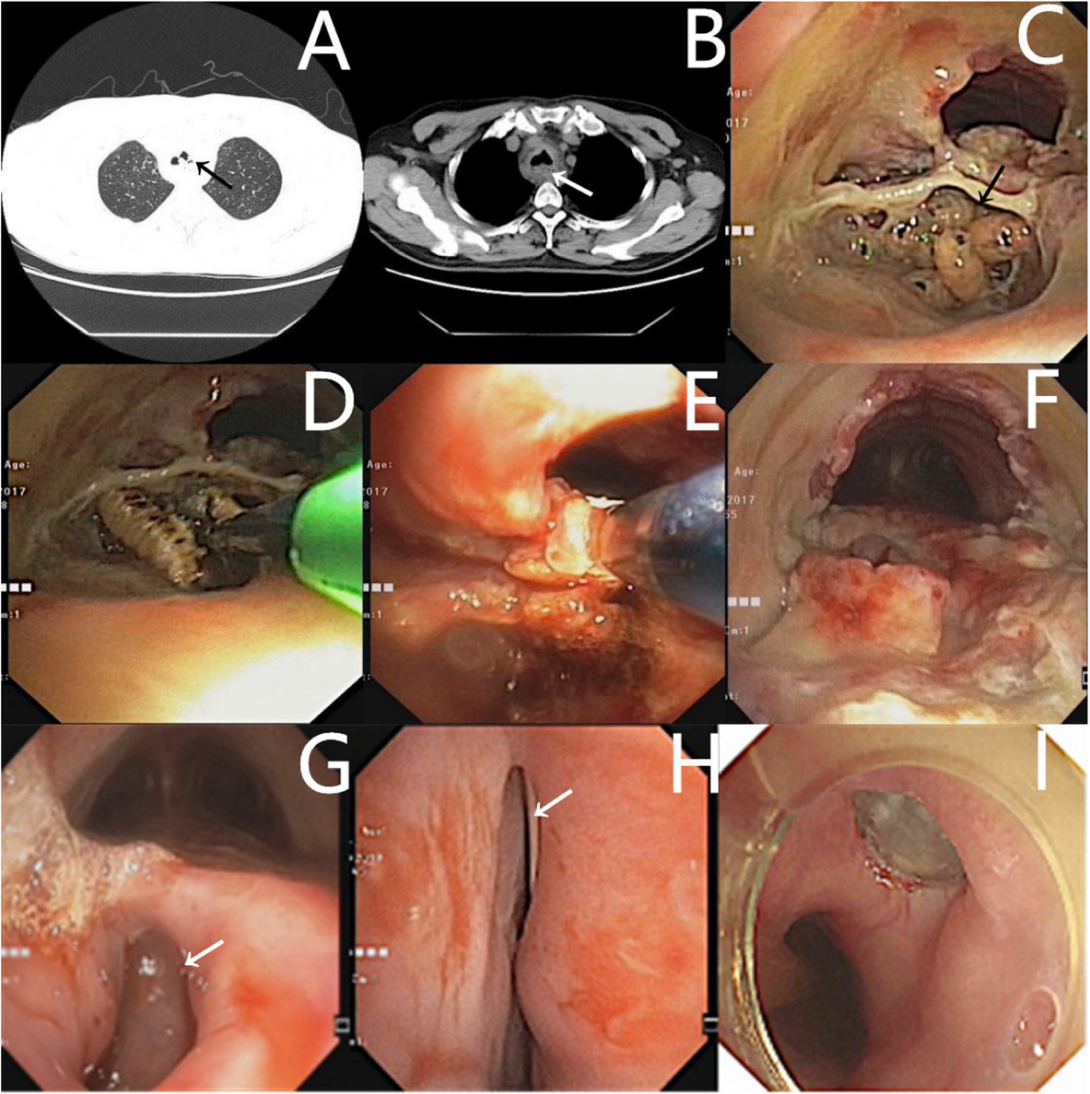
**a**, **b** A hyperdense mass involving the trachea (black arrow) and blurred fat space around the oesophagus and trachea (white arrow). **c** The first fibre-optic bronchoscopy showed a larval burrow with live larvae (black arrow) in the lower trachea, and tracheal stenosis. **d** The tracheostenosis was resolved by using electrocautery to burn the larvae, and making linear and circular incisions into the larval burrow. **e** The larvae were removed using cryotherapy and forceps, and the basal granulation was cleared. **f** The second fibre-optic bronchoscopy revealed granulation and necrosis covering the trachea, with no burrow or live larvae. The tracheal stenosis had improved. **g**, **h** The third fibre-optic bronchoscopy showed a tracheal-oesophageal fistula in the lower trachea (white arrow), and the indwelling gastric tube under the fistula (white arrow). **i** Follow-up gastroscopy performed 12 months after the procedure showed incomplete healing, with the tracheal-oesophageal fistula covering the stent

Computed tomography of the chest showed a hyperdense mass involving the trachea (Fig. 1a, arrow). The peri-esophageal and peri-tracheal fat spaces were blurred (Fig. 1b, arrow). Bronchoscopy confirmed the presence of live larvae in the left lower tracheal segment, with fibrous granulation and tracheal stenosis. Furthermore, heavy granulation was found in the upper and middle thirds of the trachea. A tracheostenosis with a smallest diameter of about 0.6 cm was noted (Fig. 1c); it was resolved using electrocautery to burn the larvae (Fig. 1d), and by making linear and circular incisions in the larval burrow and removing the larvae using cryotherapy and forceps (Fig. 1e) and clearing the basal granulation. This procedure resulted in improvement of the tracheal stenosis. Bronchoscopy showed that the left and right main bronchi and their upper and lower segments were patent. One week later, follow-up fiberoptic bronchoscopy showed granulation and membranous necrosis in the upper and middle trachea. The larval burrow was absent from the left lower trachea, but the site was covered with granulation and necrotic tissue. The trachea was restored to three-quarters of its normal size. We removed the necrotic tissue and granulation using forceps and freezing (Fig. 1f).

After the procedure, the patient experienced cough after taking food. Follow-up bronchoscopy showed a tracheal-esophageal fistula of about 1.5 cm (Fig. 1g and h) in the lower trachea, 2 cm above the carina, so an indwelling gastric tube was placed which was visible on bronchoscopy passing through the tracheoesophageal fistula. The thoracic surgeon recommended conservative medical treatment, However, the patient continued to cough up food so bronchoscopy was performed 1 month later which showed that the tracheal-esophageal fistula had not properly healed. Next, a Y-type tracheal silicone stent was placed. The patient’s condition has been stable since that time. Follow-up gastroscopy performed 12 months after the procedure showed that the tracheal-esophageal fistula had not healed and covered the Y-type tracheal silicone stent (Fig. 1i), for which we suggested surgical repair.

## Discussion and conclusions

In this case, intratracheal myiasis resulted from invasion of the trachea by dipterous larvae. Few cases of bronchial myiasis have been reported [1]. A search of PubMed using the keywords ‘human intratracheal myiasis’ or ‘tracheopulmonary myiasis,’ or ‘bronchial myiasis’ revealed five reports of five cases published up to July 2018 (Table 1). The cases were sporadic, without age or sex predilection, and all occurred in tropical and subtropical regions where flies are prevalent following endotracheal intubation or tracheotomy [3–7].

**Table 1:** Reported cases of intratracheal myiasis

Authors and year	Patient sex/age	Country	Concomitant diseases	Involved areas	Confirmative evidence	Clinical manifestations
Cornet et al. 2002	Female/60 years	US	No	Intratracheal	Cough out	Cough, haemoptysis
Cecchini et al. 2012	Male/47 years	France	Diabetes, septic shock	Left main bronchus	Bronchoscopy	No
Arindom et al. 2015	Male/13 years	Oman	No	Right upper bronchus	Bronchoscopy	Cough, dyspnoea
Ahadizadeh et al. 2015	Male/53 years	US	Cardiac arrest	Intratracheal	Inside tracheal catheter	No
Ince et al. 2015	Female/8 months	Turkey	N/A	Intratracheal	N/A	N/A

*N/A* not accessible

Notably, the case reported here is the only case with clear evidence of intratracheal colonization; furthermore, intratracheal myiasis followed by tracheal-esophageal fistula has not, to the best of our knowledge, been reported previously. The patient in this case underwent tracheotomy for nasopharyngeal carcinoma. The invasion was due to a female fly laying eggs in the main trachea through the tracheotomy. Tracheostomal myiasis has also been reported outside of China [8–10]. The open space formed by the tracheotomy and the odor emitted by the secretions at the fistula are important contributing factors. In addition, poor personal hygiene and the postoperatively suppressed cough reflex provide favorable conditions for intratracheal colonization. The treatment of myiasis involves surgical removal of larvae and administration of the broad-spectrum antiparasitic drug ivermectin [4]. For the current case, surgical treatment was feared to be too traumatic in the face of severe airway stenosis, so fiberoptic bronchoscopic treatment was initially attempted. The larvae were carefully removed from the surface of the burrow using tracheoscope forceps, taking care not to drop them into the lower airway; unfortunately, more maggots hatched and crawled out afterwards. Bronchoscopy revealed different sizes of maggots in the airway including 0.1 mm living and peristaltic larvae in a tracheal tissue sample. To completely remove larvae and reduce the risk of local scar formation leading to tracheal stenosis, electrocautery and cryotherapy were performed. Follow-up bronchoscopy was used to assess recovery and treatment complications (such as scar re-stenosis and tracheal-esophageal fistula). Postoperatively, the patient continued to cough up food. A tracheal-esophageal fistula was observed by fiber-optic bronchoscopy, and was considered a result of larvae damaging the tracheal mucosa. Medical treatment was considered, given that the patient was in poor physical condition and probably intolerant of surgery. The diameter of the fistula was 1.5 cm; biogel therapy and chemical cauterization for fistulas of this size often fails [11, 12]. Therefore, airway and/or esophageal stenting was considered. Shin et al [13] reviewed stenting methods for tracheal-esophageal fistulae of various causes and types, which consist of: (1) esophageal stenting for tracheal-esophageal fistulae combined with severe esophageal stricture but very mild, if any, tracheal stenosis; (2) tracheal stenting when the esophagus and trachea are without remarkable stenosis; (3) tracheal stenting for severe tracheal stenosis without esophageal stenosis; and (4) esophageal and tracheal stenting for concomitant severe esophageal and tracheal stenosis. As the patient described here had no significant esophageal or tracheal stenosis, coated graft stenting was preferred. Finally, bronchoscopic tracheal stenting and indwelling gastric catheterization were performed. At 20 months post-op, the fistula had not healed and the stent remained to prevent reflux.

In this patient, the maggots were located at the junction between the lower end of the tracheal cannula and the trachea. We speculate that the maggots had been present for several weeks and had gnawed through the tracheal and esophageal walls, resulting in tracheostomal hemorrhage. The maggots climbed up to the tracheal wound after the tracheal cannula was removed. If not properly treated in a timely manner, intratracheal myiasis may cause severe complications, including secondary infection, respiratory failure [5], tracheal stenosis, and tracheal-esophageal fistula. The prognosis is generally good when no complication occurs. Prevention is feasible, particularly post-tracheostomy, in older patients of low socio-economic status, those living in unsanitary environments, those who abuse alcohol, patients with central nervous system conditions, and those with poor personal hygiene [14].

In conclusion, tracheal-esophageal fistula is a serious complication of endotracheal myiasis and is difficult to treat. Tracheal stenting can improve the resulting reflux but cannot treat the condition. For post-tracheostomy patients, healthcare providers and caregivers should pay attention to the care and monitoring of wounds and maintenance of a tidy, clean living environment to prevent intratracheal myiasis.

## Data Availability

The materials described in the manuscript are available from the corresponding author on reasonable request.

## Abbreviations

cm: centimetre
g: gramma
L: Liter
